# Quality Control of Traditional Cannabis Tinctures: Pattern, Markers, and Stability

**DOI:** 10.3390/scipharm84030567

**Published:** 2016-04-18

**Authors:** Wieland Peschel

**Affiliations:** Centre for Pharmacognosy and Phytotherapy, Department for Pharmaceutical and Biological Chemistry, The School of Pharmacy, University College London, 29-39 Brunswick Square, London WC1N 1AX, UK; wieland.peschel@ema.europa.eu; Tel.: +44-20-3660-7742

**Keywords:** *Cannabis sativa* L., traditional tinctures, stability (HPLC, THC, CBN, CBG)

## Abstract

Traditional tinctures of *Cannabis sativa* L. became obsolete before elucidation of the main cannabinoids and routine quality testing for medicines. In view of increasing medicinal use of cannabinoids and associated safety concerns, tinctures from a Δ9-tetrahydrocannabinol (THC)-type chemovar were studied. High-performance liquid chromatography with diode-array detection (HPLC/DAD) was used to determine THC, Δ9-tetrahydrocannabinolic acid A (THCA), cannabinol (CBN), cannabidiol (CBD), cannabidiolic acid (CBDA), cannabigerol (CBG), cannabigerolic acid (CBGA), cannflavin A/B, and total phenolics. Derived group and ratio markers describe absolute and relative profiles when varying plant part (flos, folium), extraction solvent (EtOH percentage), storage conditions (‘shelf’ or ‘fridge’ up to 15 months), and pasteurization (2 h 70 °C, 20 min 80 °C). Tinctures from female flowering tops contained ten-fold more cannabinoids than tinctures from leaves; tinctures (80%–90% EtOH) contained ten-fold more cannabinoids than tinctures (40% EtOH). The analysis of CBGA + CBG, the main co-cannabinoids aside from THCA + THC, appears more relevant than CBDA + CBD. The decarboxylation of THCA to THC—the main change during storage of freshly prepared tinctures—is after 15 months in the ‘fridge’ comparable to 3 months on the ‘shelf’. Minimally increased CBN totals did not correlate to diminished totals of THCA and THC (up to 15% after 3 months ‘shelf’, 45% after 15 months ‘fridge’). Instead, total cannabinoids or acidic/neutral cannabinoid ratios are better stability markers. Moderate changes after pasteurization and partial losses below 10% for total cannabinoids after 9 months ‘fridge’ indicate possibilities for a reasonable shelf life. Yet storage and use of non-stabilized tinctures remain critical without authorized specification and stability data because a consistent cannabinoid content is not guaranteed.

## 1. Introduction

In the 19th century, the Irish surgeon O’Shaughnessy reintroduced the use of cannabis from India into Western medicine [1,2]. *Cannabis sativa* L. (Cannabaceae)—largely in the form of ethanolic tinctures—was then part of main Western pharmacopoeias until the 1930s, when it was removed gradually with increasing recreational use and its legal prosecution [3]. In the UK for instance, cannabis disappeared from the 1932 British Pharmacopoeia but remained in the 1949 British Pharmaceutical Codex (BPC) and was thus available for use via prescription until 1971 in form of the substance (Cannabis BPC), an extract (ethanolic percolate), and a ‘tincture’ (dilution of cannabis extract BPC) [4]. The standardization included the ethanol content but not the cannabinoid content since the main cannabinoids were elucidated only in the 1960s. Cannabis analysis, as established in the 1970s and 1980s, has been focusing since then predominantly on forensic purposes, but not on the standardization of traditional preparations used for decades.

The current medicinal use of cannabis, whether it is authorised, tolerated, under discussion, or strictly forbidden, is associated with doubts on the safe use due to uncertainty factors including dosage and route of administration [5,6]. The inhalation of natural cannabis (smoking or inhalators) provides a rapid and relatively controllable onset compared to the slow and less predictable bioavailability of main cannabinoids when taken orally [7]. Likewise, for an authorized medicinal product with cannabis extract as the active substance, a buccal spray circumvents gastrointestinal metabolism with absorption mainly over the oral mucosa [8,9]. The generally high risk of cannabis overdose with unwanted side effects was found more frequently from oral ingestion of non-standardized preparations such as cookies [10]. Nonetheless, the consistent content of traditional tinctures and its analysis is not only of historic interest—as they remain somewhat popular particularly for non-smokers—but possibly because of its simple preparation and traditional oral use. While individual variation in pharmacokinetic and pharmacodynamic response requires medical monitoring, chemical variability adds to safety concerns. Depending on the legal status, in some countries the availability of standardized preparations for oral use was identified as a tool for risk minimization and risk management. Over 10 years ago, the Dutch ‘Bureau voor Medicinale Cannabis’ issued the first modern monograph in line with standard pharmacopoeia structure for *Cannabis flos* with Δ9-tetrahydrocannabinol (THC), cannabidiol (CBD), and cannabinol (CBN) specified via high-performance liquid chromatography with diode-array detection (HPLC-DAD) [11]. Consistent quality via controlled production of defined chemovars and standardization has been introduced alongside regulated distribution channels via medical prescriptions and pharmacies with information of patients and health care professionals [12]. However, the specific quality of traditional liquid extracts has not been investigated so far, although the knowledge about its variable content is essential for risk assessment and regulation nowadays.

Within standard quality control, the stability of medicinal products with natural compound mixtures is particularly critical since it determines shelf-life, storage conditions, and analytical expenditure to meet regulatory requirements with regards to appropriate stability-indicating markers. The aim of this study was to investigate the range and variability of potential analytical markers in benchmark samples. Hypothesizing that traditional tinctures have been prepared with non-standardized herbal drugs—with variable proportions of flowers and leaves as well as variable alcohol strength—tinctures of different EtOH strength from the resin-rich female inflorescences (‘*Cannabis flos*’) were compared versus lower leaves (‘*Cannabis folium*’) that are found as admixtures in marijuana drugs. Previously described, validated HPLC-DAD methods were used to profile extracts that are prepared without heat and characterized by a high content of cannabinoid acids [13]. Group markers were summarized from the determination of the main cannabinoids (THC, CBD, cannabigerol (CBG)), their corresponding original acidic counterparts (tetrahydrocannabinolic acid (THCA), cannabidiolic acid (CBDA), and cannabigerolic acid (CBGA)), cannabis-specific prenylated flavones (cannflavin A (CFL-A) and B (CFL-B)), and other phenolic constituents (flavonoids such as orientin, vitexin, or caffeic acid derivatives). Beyond quality control, such simplification of the complex cannabis chemistry into analytical sum parameters can have relevance regarding safety/efficacy as previously shown for the relation of total cannabinoid content and viability reduction in cancer cells (Table 1) [13]. Traditional preparation and storage was simulated; i.e., simple macerates were stored under ‘real world’ conditions: amber glass containers with an air layer stored either on a shelf, i.e., at room temperature with certain daylight exposure, or in the fridge in the dark. In addition, the effect of short-term heating was tested, which is often a necessary step during manufacturing.

## 2. Materials and Methods

### 2.1. Plant Material

The seeds of the *Cannabis sativa* L. variety ‘northern lights 5 crossed with haze’ were purchased from Pukka Seed Company (Guildford, UK). The chemovar popular in illicit plant use for recreational purposes belongs to the THC-type (>20%), but has no official specification or standardization. This cultivar was grown in the laboratory greenhouse as previously described [14] and female plant samples harvested after 4 months were split into flowering tops, lower leaves, and roots. Stalks >3 mm diameter were removed. Flowers and roots were cut shortly before extraction, while one part of the lower leaves were ground using a mill (MF-10 basic, IKA-Werke, Staufen, Germany) to obtain a powder with maximum 0.2 mm particle size. The four drugs were stored at room temperature in the dark and used after 5 or 24 months before extraction.

### 2.2. Reference Standards

Phytocannabinoids were purchased from THC Pharm GmbH (Frankfurt, Germany) including Δ9 tetrahydrocannabinol (THC), cannabidiol (CBD), cannabigerol (CBG), cannabinol (CBN), and Δ9 tetrahydrocannabinolic acid A (THCA). All samples were stored in the dark at −20 °C. Reference standards were cannflavin A, cannflavin B, (CFL-A/CFL-B, kindly provided by Giovanni Appendino, Novarra, Italy), olivetol (Sigma, Dorset, UK), furthermore luteolin, kaempferol, apigenin (all Extrasynthese S.A. Co., Genay-Sedex, France), quercetin (Sigma) orientin, homorientin, vitexin, isovitexin (all Extrasynthese S.A. Co.), and rutin (Sigma), as well as chlorogenic acid and caffeic acid (both Sigma). Cannabinoids were kept stable when stored at −20 °C in brown glass vessels in the dark. Deterioration could be first detected after 24 months of storage at −20 °C and occasional brief exposure to room temperature and light for experiments. CBN was the major impurity of THC with a percentage of 0.66% of the THC peak (HPLC profile AUC at 214 nm). Two other less concentrated unidentified impurities with a characteristic cannabinoid spectrum were detectable at retention times of 31.77 min and 35.59 min. The sum of impurities was below 2% (1.58%). CBD showed three additional peaks with a sum percentage of 0.83% of the CBD AUC (214 nm) at 17.23, 17.95, and 19.98 min. Cannflavin A showed another flavonoid (t_R_ = 20.18; 4.61%) and one peak similar to cannflavin B (t_R_ = 12.46; 4.14%). For CBN and CBG, no instabilities were detectable at applied storage conditions.

### 2.3. Preparation of Hydroethanolic Tinctures

#### 2.3.1. First Batch—Flower Tinctures (20%/40%/80% EtOH)

Tinctures (ratio between drug and extraction solvent 1:10) were prepared using the 5-month-old starting material: 10 g were macerated with 100 mL of different mixtures EtOH/water (20%, 40%, and 80% *v*/*v*) for three days at room temperature in the dark under agitation (aluminum foil-covered 500 mL Erlenmeyer flask on an automated shaker). After filtration, the tinctures were split into various vials, stored at −20 °C, and thawed directly before chemical analysis (abbreviated as: E20, E40, E80).

#### 2.3.2. Second Batch—Flower Tinctures and Leaf Tinctures (40%/60%/90% EtOH)

The same starting material—but two years old—served as a comparison of flowering tops (abbreviated as: F40, F60, F90) with lower leaves (abbreviated as: L40, L60, L90). Tinctures (drug-solvent ratio 1:10) were prepared by maceration using 20 g samples and were macerated with 200 mL of different mixtures EtOH/water (40%, 60%, and 90% *v*/*v*) for two days at room temperature in the dark (aluminum foil-covered 500 mL Erlenmeyer flask). In addition, cold water macerates (3 days, CW) and a hot water infusion (100 °C cooling down for 1 h, HW) of the same materials as well as 60% EtOH tinctures from the ground leaf (particle size less 0.2 mm = GL60) and the cut root (R60) of the plants were prepared. After filtration, the macerates/infusions were split into various vials and stored at 4 °C in the dark. The chemical profile of the fresh preparations was determined within 48 h of preparation.

### 2.4. HPLC Analysis

Two methods previously described and validated were applied [13]. The overall fingerprint (detection at 214, 254, 275, and 324 nm) allowed us to address the ratio between cannabinoids (CAN_tot_) and other phenolic compounds (TPC), while the cannabinoid profile method (λ = 214 nm) served for the quantification of cannabinoids and cannflavins. The HPLC Waters™ system 900, with a Waters™ 996 PDA detector and a Waters™ 717 plus autosampler device and Millenium or EmPower software were used equipped with an Ace^®^ 5 Phenyl (25 cm × 4.6 mm) column (ACT, Aberdeen, UK) for the fingerprint and an Agilent Zorbax RX-C18 column (5 µm 4.6 × 250 mm Highchrom, Reading, UK) and a Nova-Pak^®^ C8 Guard Column 3.9 × 20 mm, 2/pkg (Waters UK Elstree, UK) for the cannabinoid profile. Gradient solvent mixtures of water (TFA 0.1%) (solvent A), a water-acetonitrile mixture (65:35, TFA 0.1%) (solvent B), and acetonitrile (solvent C) were used for the fingerprint (80 min including pre- and washing phase). Solvent B and C were used for the cannabinoid profile (55 min including pre- and washing phase). Reference standards were used as described previously, while liquid extracts were injected directly or after 1:1 dilution, in case of highly concentrated tinctures.

### 2.5. Determination and Calculation of Single, Group, and Ratio Markers

#### 2.5.1. Peak Identification and Qualification

**Figure 1:**
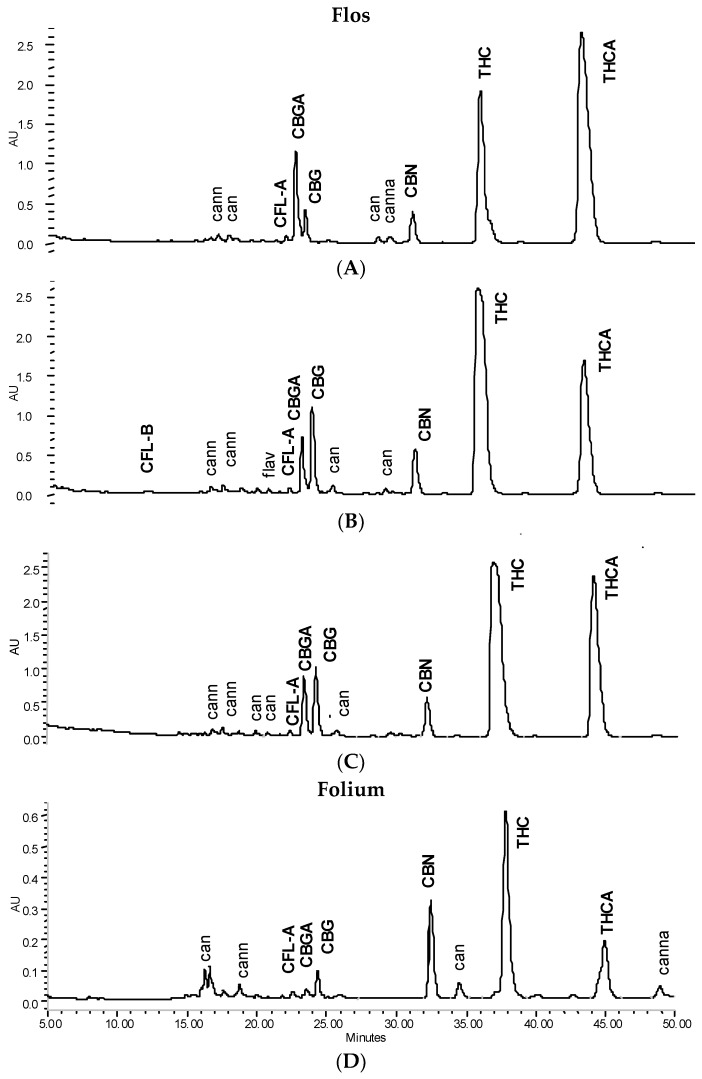
HPLC cannabinoid profile (λ = 214 nm) of 90% tinctures from flowering tops or leaves. (**A**) Freshly prepared; (**B**) after 3 months at room temperature/daylight (‘shelf’); (**C**) after 9 months at 4 °C in the dark (all from flowering tops); (**D**) after 9 months at 4 °C in the dark from leaves. Cannabigerol (CBG); cannabigerolic acid (CBGA); cannabinol (CBN); cannflavin A (CFL-A); cannflavin B (CFL-B); Δ9-tetrahydrocannabinol (THC); Δ9-tetrahydrocannabinolic acid A (THCA).

**Figure 2:**
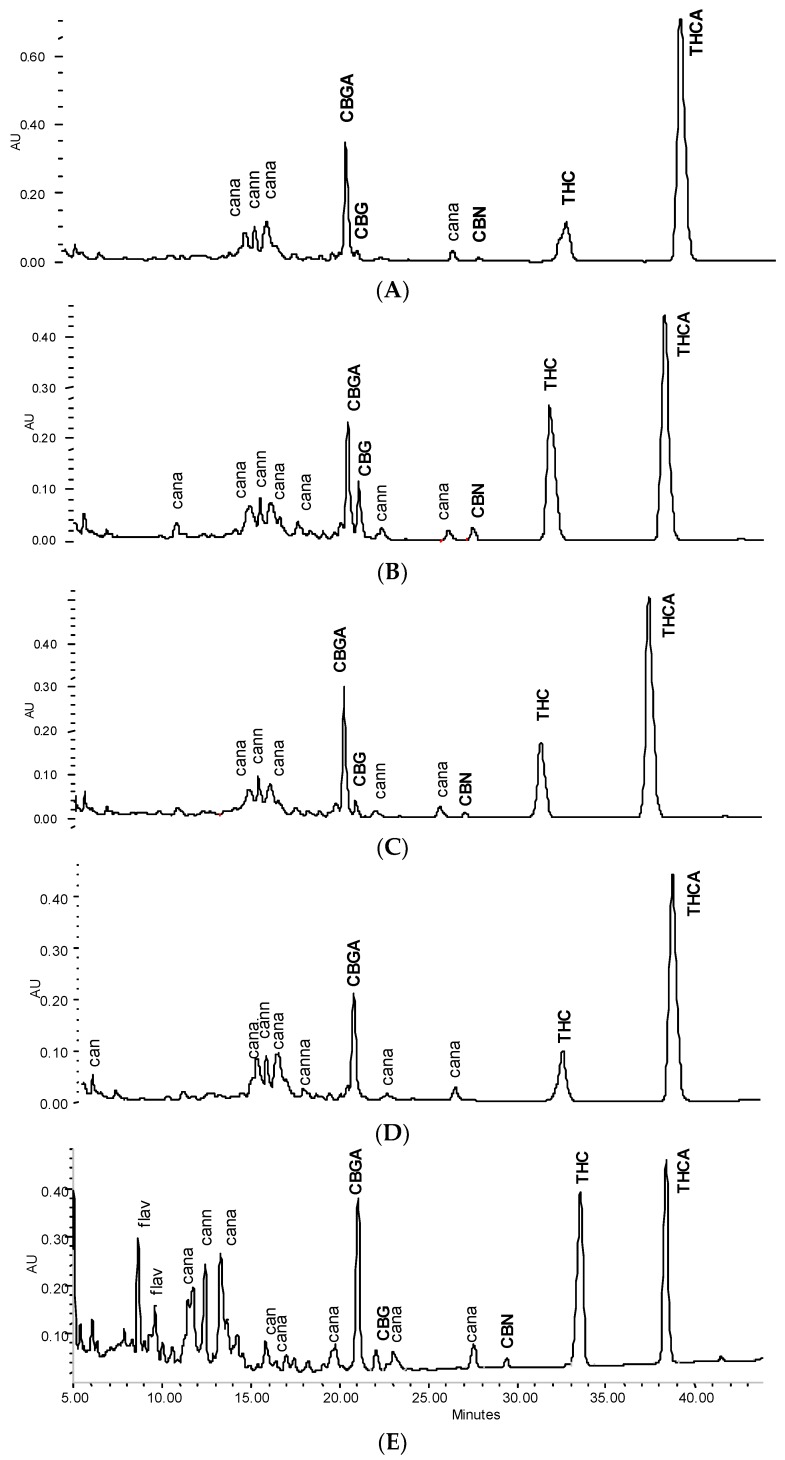
HPLC cannabinoid profile of a 40% tincture from flowering tops (λ = 214 nm). (**A**) Fresh; (**B**) +3 months ‘shelf’; (**C**) +3 months ‘fridge’; (**D**) and 2 h heating at 70 °C; (**E**) +15 months ‘fridge’.

In line with the previously described method, peaks were identified using standard references [13]. Non-identified peaks were qualified by the PDA spectrum and allocated to the following groups: flavonoid, phenolcarbonic acid (summarized total phenolic content TPC), neutral cannabinoids (CAN), and cannabinoid acids (CANA) for the calculation of group markers (see Table 1). For identification purposes (not relevant for calculation here), it is further possible to differentiate between neutral cannabinoids with a THC/CBD-type spectrum and those with a CBN/cannabichromene (CBC) type spectrum as well as acidic cannabinoids with a THCA/CBDA-type spectrum and those with a CBNA/CBCA type spectrum as previously described [13,15]. Non-identified qualified peaks are allocated in chromatograms (Figure 1 and Figure 2) as: can = neutral cannabinoids with THC/CBD-type spectrum, cann = neutral cannabinoids with CBN/CBC-type spectrum; cann = acidic cannabinoids with THCA/CBDA-type spectrum, canna = acidic cannabinoids with CBNA/CBCA-type spectrum; flav = flavonoids.

#### 2.5.2. Assay

The concentration of THC, THCA, CBD, CBDA, CBG, CBGA, CBN, CFL-A, and B was determined by means of external standards using the validated cannabinoid profile method as previously reported [13]. The concentration of non-identified qualified cannabinoids was calculated as THC. Spectra-qualified flavonoids and phenolcarbonic acids were calculated from the fingerprint method as vitexin and chlorogenic acid. All measurements were performed in triplicate and expressed as means ± SD.

#### 2.5.3. Group and Ratio Marker Calculation

The group markers THC_tot_, CBD_tot_, CBG_tot_, CFL as well as CAN (neutral cannabinoids), CANA (acidic cannabinoids), CAN_tot_ (total cannabinoids), and oCAN (other cannabinoids than THC_tot_, CBD_tot_, CBG_tot_,) were summarized from single determinations of identified and qualified peaks based on the cannabinoid profile method. Total phenolics (TPC) were summarized from flavonoids and phenolcarbonic acid determination using the fingerprint method (Table 1). Group markers allowed for further calculation of relevant ratios such as THC_tot_/(CBG_tot_ + CBD_tot_), CANA/CAN, and CAN_tot_/TPC (Table 1).

### 2.6. Stability Tests

The tinctures (EtOH 40%/60%/90% flower and leaf tinctures) were split and 40 mL portions poured into amber glass containers and stored in the fridge (2–6 °C) or on a shelf at room temperature in a room with daylight, but without direct sunlight exposure simulating standard ‘shop’ or ‘home’ storage conditions on a shelf at temperatures around 20 °C. The profile of tinctures was first tested fresh after preparation (24/48 h storage in the dark at 2–6 °C) and retested after 3 months for fridge and room temperature variants. The room temperature storage was not further continued while the fridge variants were extended and retested after 9 and 15 months.

For investigation of the effects of short-term heating, 5 mL of selected ‘fridge samples’ of the 40%/60%/90% tinctures were heated in closed containers either at 80 °C for 20 min or at 70 °C for 2 h simulating pasteurization conditions. After cooling to room temperature, they were analysed the same day.

### 2.7. 3-(4,5-Dimethylthiazol-2-yl)-2,5-diphenyl Tetrazolium Bromide (MTT) Assay

The Hela-*luc* (Hela-IL-6) cell line was kindly provided by Dr. M. L. Lienhard Schmitz, University of Giessen, Germany). Hela-*luc* were maintained in Dulbecco’s Modified Eagle’s Medium (DMEM) (Invitrogen, Paisley, UK) supplemented with 10% fetal bovine serum and antibiotics at 37 °C in a 5% CO_2_ humidified atmosphere and split when confluent.

The classic MTT assay measures mitochondria viability and was used here as an indicator of cytotoxicity. Briefly, cells were incubated at 37 °C and 5% CO_2_ atmosphere for 24 h in media supplemented with PBS, at different concentrations up to 100 μL/mL. Controls received the vehicle and corresponded to 100% viability. The yellow water-soluble substrate MTT was converted by living cells into a water-insoluble blue formazan product. The coloured metabolite was dissolved in DMSO and measured using an Anthos Lucy 1 luminometer/photometer (Anthos-Biochrome Ltd., Cambridge, UK) at 490 nm, and the data were collected and processed by using Stingray 1.5 (Dazdaq Ltd., Brighton, UK). Corrected absorbance values (adjusted to blank absorbance—no MTT) were converted into % growth values in comparison to the non-treated control. Viability less than 85% of the control were considered as toxic and expressed as maximum non-toxic concentration (MNTC = 85% of respective control). Tests were performed in duplicate (two samples per plate, two plates per experiment), and each sample was tested in three independent experiments expressed as means (± standard error of the mean—SEM, *n* = 3). Tincture samples were tested when fresh and after 6 months storage at −18 °C in the dark.

### 2.8. NF-κB IL-6/Luciferase Assay

HeLa-*lu*c cells are stably transfected with a luciferase reporter gene controlled by the interleukin (IL-6) promoter. The plasmid upstream from a luciferase gene, together with the selection plasmid pMEP4 includes the recombination signal sequence binding protein Jkappa, which is constitutively bound to the NF-κB site of the IL-6 promoter and acts as a negative regulatory factor. IL-6 is one of the target genes for activated NF-κB. Therefore, the luciferase produced can be measured by IL-6 dependently, signalling activation or inhibition of NF-κB. Tests were performed as previously described [14]. In brief, cells were exposed to tincture samples (final concentrations in the media 0.002–25 μL/mL) for 40 min before the stimulant phorbol myristate acetate (PMA, 50 ng/mL) was added. The cells were incubated (37 °C) for 6 h before lysis, transfer, and automatic addition of the luciferase substrate (Promega, Southampton, UK) using a luminometer/photometer (Anthos Lucy 1) and the resulting luminometric reading recorded following a reaction time of 10 s. Positive (stimulated cells without sample) and negative (resting cells without stimulation) controls were included. Means were calculated from two readings per sample, two samples per plate, two plates per experiment, and two independent experiments. SEMs were calculated for the latter two (*n* = 4). Tincture samples were tested when fresh and after 6 months of storage at −18 °C in the dark.

## 3. Results

### 3.1. Main Pattern and in Vitro Activity of Tinctures—Influence of the Solvent

**Figure 3:**
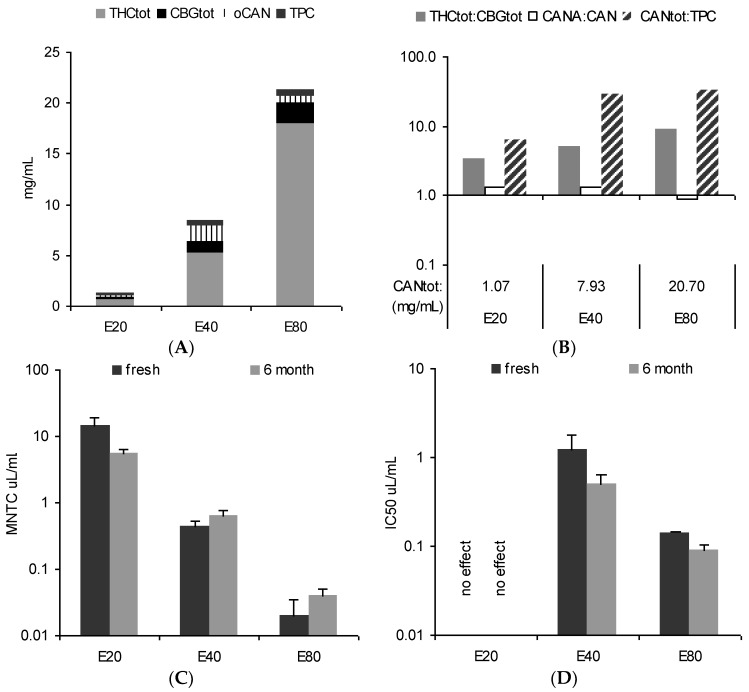
Profile of fresh tinctures (20%, 40%, 80% EtOH) from cannabis flowering tops. (**A**) Absolute content in THC_tot_, CBG_tot_, other cannabinoids (oCAN), and phenolics (TPC); (**B**) total cannabinoid content (CAN_tot_) with relative ratio markers THC_tot_/CBG_tot_, CANA/CAN, and CAN_tot_/TPC (see Table 1); (**C**) maximum non-toxic concentration (MNTC) in HeLa cells (MTT 24 h, mean ± SEM, *n* = 3); (**D**) inhibition of stimulated NF-κB activation (phorbol myristate acetate (PMA) 6 h, mean ± SEM, *n* = 4). Neutral cannabinoids (CAN); cannabinoid acids (CANA); total cannabinoids (CANtot); cannabigerol + cannabigerolic acid (CBGtot); Δ9-tetrahydrocannabinol + Δ9-tetrahydrocannabinolic acid A + cannabinol (THCtot); total phenolic content (TPC).

The tincture profiles from 5-month-old comminuted female flowers (E20, E40, and E80) are differently illustrated in Figure 3A,B: (A) the total cannabinoid content (bar height) is split into portions of Δ9-tetrahydrocannabinol + Δ9-tetrahydrocannabinolic acid A + cannabinol (THC_tot_), cannabigerol + cannabigerolic acid (CBG_tot_), and other not identified cannabinoids (oCAN) and completed with total phenolic content (TPC) values. Cannabidiol + cannabidiolic acid (CBD_tot_) was below the limit of quantification (LoQ); (B) A summarizing profile illustrates key information about the herbal preparation: the total cannabinoid content (CAN_tot_) as an overall ‘strength marker’ in numerical form in the abscissa and the ratios for THC_tot_/CBG_tot_ (ratio main compound groups as chemotype indicator), CANA/CAN (decarboxylation indicating age/storage), and CAN_tot_/TPC (plant part and polarity indicator) as bars using logarithmic scaling (abscissa set at 1).

Expectedly, the total amount of THC and the ratio to co-constituents is influenced by the EtOH strength. Here, compared to a traditional 80% EtOH tincture, only 1/3 (1/20) of the cannabinoids were extracted using 40% EtOH (20% EtOH), respectively. Nonetheless, THC-dominant tinctures are always obtained with traditional one-step extraction from THC-type material - even with rather polar hydroethanolic solvents. From a qualitative perspective, in principle the same characteristics indicate the equivalent drug source, i.e., THC type and dominance, comparable status of decarboxylation, and low TPC values. Nevertheless, it also shows some potentially activity-relevant relative differences such as a ten-fold amount of THC_tot_ over CBG_tot_ in E80 which is only about three-fold in E20 and E40.

The in vitro pharmacological profile in HeLa cells (cytotoxicity indicated by mitochondria viability measured after 24 h and potential anti-inflammatory and cell fate-relevant effects measured via inhibition of phorbol myristate acetate (PMA)-stimulated NF-κB activation after 6 h) reflected chemical differences according to the extraction solvent (Figure 3C,D). Interestingly, repeated tests after 6 months showed some changes over time, although tinctures were stored at −18 °C in the dark. This encouraged testing for chemical profile changes with a new batch of tinctures when stored under common practical conditions, i.e., at room temperature or in ‘fridge’ conditions.

### 3.2. Profile of Leaf and Flower Tinctures According to Storage Conditions (3 Months)

**Figure 4:**
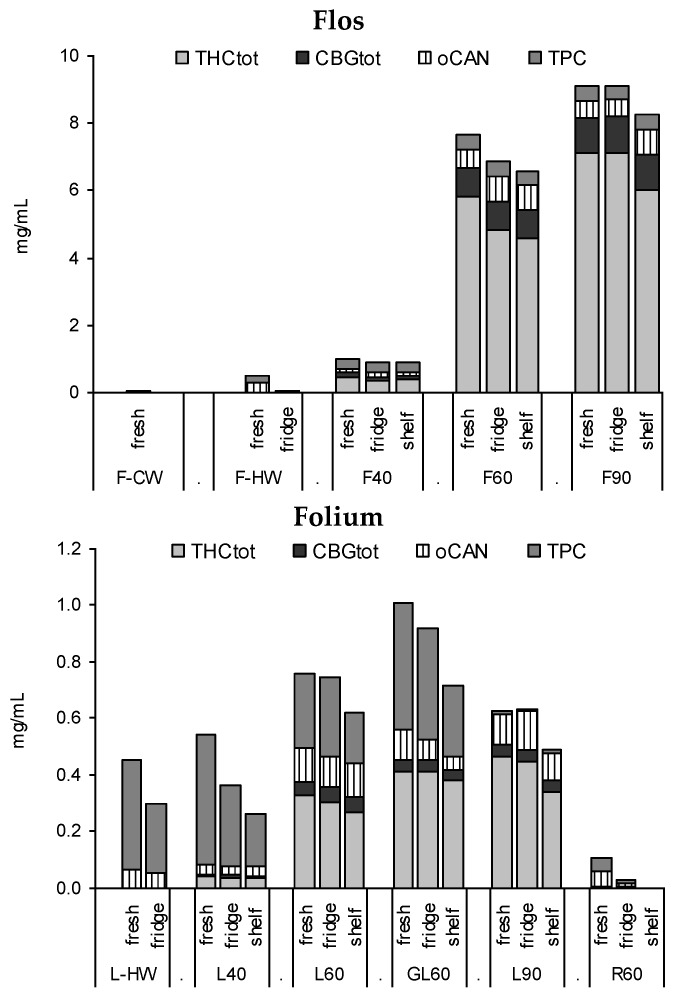
Profiles of tinctures from flowering tops (F), leaves (L, GL), or root (R) fresh and after 3 months of ‘fridge’ (4 °C/dark) or ‘shelf’ (15–25 °C/light). CW, cold water macerate; HW, hot water infusion, 40%, 60%, and 90% EtOH macerates.

Tinctures from flowering tops (F40, F60, F90) and leaves (L40, L60, GL60, L90) extracted from the 2-year-old drug diverged mostly in the quantity of the extracted compounds alongside some qualitative differences in flavonoid and cannabinoid profiles. Root extracts (R60) as well as cold water macerates (CW) do not appear suitable for cannabinoid or flavonoid extraction, while with hot water a considerable amount of phenolics are extracted from cannabis leaves, but few cannabinoids are (Figure 4). Notable is the increased yield (THC_tot_ and TPC) for the leaf drug with lower particle size (GL60) compared to the comminuted drug L60 as well as the substantial quantitative discrepancy of F40 compared to F60 or F90 (Figure 4).

**Figure 5:**
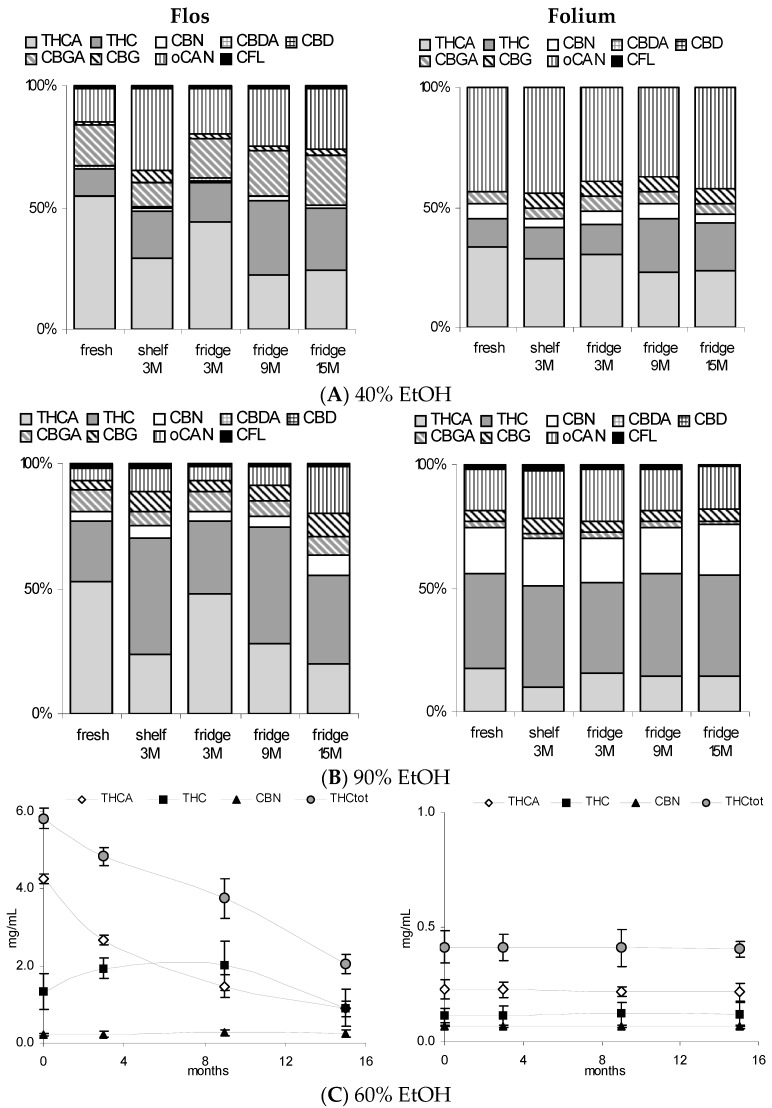
Comparison of tincture profiles from Cannabis flos vs. folium. (**A,B**): Relative cannabinoid profile of 40% and 90% tinctures (Δ9-tetrahydrocannabinolic acid A THCA, Δ9-tetrahydrocannabinol (THC), cannabinol (CBN), cannabigerolic acid (CBGA), cannabigerol (CBG), other non-identified cannabinoids oCAN, and cannflavins CFL after 2 days, 3 months (15–25 °C/light), 3, 9, and 15 months (4 °C/dark) storage; (**C**) Absolute values for THCA, THC, CBN, and their sum (THC_tot_) in 60% tinctures from flowering tops and leaves (ground) over 15 months (4 °C/dark). Mean of measurement in triplicate ± SD. Cannabidiol (CBD); cannabidiolic acid (CBDA).

Tincture aliquots stored in amber glass bottles either at 4 °C (fridge/dark) or 20 °C (moderate light exposure) were re-analysed after 3 months (Figure 1, Figure 2 and Figure 4). In F40 there was a 20% decrease of CAN_tot_ at room temperature (RT) and 10% under cool conditions. Moderate losses in CAN_tot_ were also visible in F60 and F90 (at RT 7.2 to 6.2 mg/mL and 8.7 to 7.8 mg/mL, respectively) and the decarboxylation was accelerated (at RT CANA/CAN-ratio: 2.20 to 0.39 and 2.78 to 0.41, respectively). While the cannabinoid profile showed only minimally increased CBN levels, the conversion of Δ9-tetrahydrocannabinolic acid A (THCA) to THC and CBGA to CBG after 3 months at room temperature/light exposure (Figure 2B) was more advanced compared to fridge/dark conditions, even after 15 months (Figure 2C,E). Surprisingly, leaf extracts that were absolutely low in CAN_tot_ remained relatively unchanged after 3 months at room temperature or even 15 months cool storage, which may signal a certain equilibrium or at least reduced decarboxylation (Figure 5).

### 3.3. Stability of Leaf and Flower Tinctures in Cool, Dark Conditions (15 Months)

**Figure 6:**
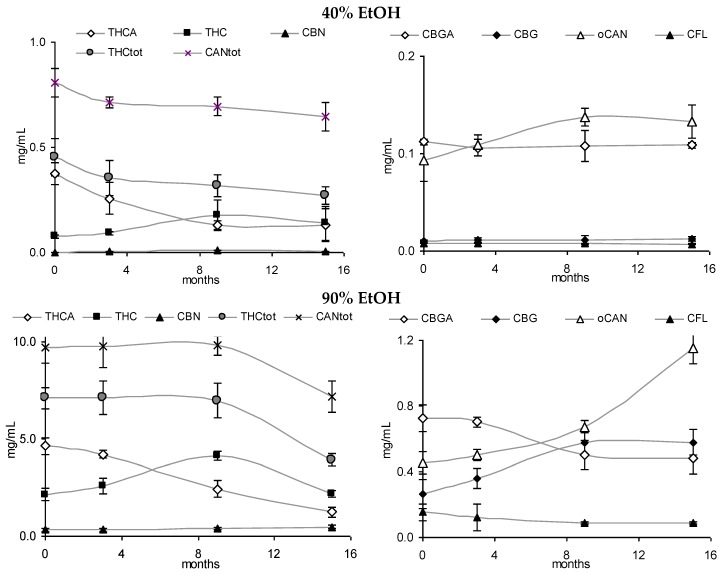
Absolute values for cannabinoids and cannflavins in 40% and 90% tinctures from flowering tops (40% EtOH, 90% EtOH) over 15 months (4 °C/dark).

After the continued cool storage (4°C/dark), samples were re-analyzed after 9 and 15 months and compared regarding relative and absolute changes. Figure 5A,B illustrates the relative change of the cannabinoid profile for 40% and 90% leaf or flower tinctures. High-cannabinoid flower tinctures more than low-cannabinoid leaf tinctures were prone to reduction of THCA (CBGA) in relation to THC (CBG). The drop in THC_tot_ vis-à-vis other cannabinoids was highest in F40, while the CBGA to CBG conversion was least progressed here. In Figure 5C, the absolute values for THC, THCA, CBN and the sum of all three (THC_tot_) are shown for 60% flower and leaf ground tinctures. The reduction of THCA and initial increase (until 9 months) and decrease thereafter of THC in F60 goes along minimally increased CBN levels that do not compensate for the overall THC and THCA reduction. The tinctures of the already ‘aged’ ground leaf starting material (low cannabinoid, low acids, relatively high CBN) did, in contrast, hardly change over 15 months. In Figure 6, absolute values for THCA, THC, CBN, THC_tot_, and CAN_tot_ as well as for CBGA, CBG, and CFL are shown for 40% and 90% flower extracts. In both cases, the decarboxylation from THCA to THC represents the main change, while losses in THC_tot_ and CAN_tot_ in particular between 9 and 15 months were higher in F90 than in F40, indicating divergent stability over time according to the solvent. Relevant changes in relation to the start value in the fresh tinctures are summarized in Table 2. Apart from those main features, the appearance of many new peaks, in particular between 5 and 20 min retention time after 15 months of storage, indicates a challenging task to qualify newly arising minor cannabinoids, but also flavonoids after 15 months of storage (Figure 2E).

### 3.4. Stability of Leaf and Flower Tinctures after Short-Term Heating

**Figure 7:**
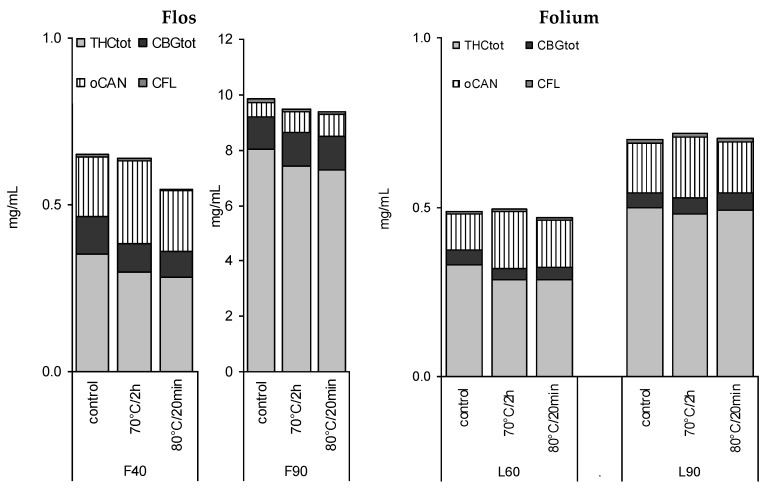
Absolute profile of 40%, 60%, and 90% tinctures from flowering tops (F) or leaves (L) after 3 months (4 °C/dark—control) and additional pasteurisation (long-term 70 °C/2 h or short-term 80 °C/20 min).

In view of previous reports on the temperature influencing cannabinoid stability, four tinctures (L60, L90, F40, F90) were heated for a short time close to the critical temperature for decarboxylation reported earlier as being between 60 °C [16] and later 90 °C [17] (Figure 7). A short time of pasteurization at 80 °C for 20 min and a longer heating period (70 °C, 2 h) showed a minor decrease in CAN_tot_ (e.g., L60 0.49 to 0.46 mg/mL), and a more significant decrease in THC and THC_tot_ in F40 tinctures, but no signs of accelerated decarboxylation. In contrast, THCA/THC and CBGA/CBG ratios seemed to be partially restored in favour of the acids, in particular, in more aqueous tinctures (Table 2). CBN levels did not increase (Figure 2D). Apart from F40, for the other tinctures, the maximum reduction of 5% to 15% of CAN_tot_ or THC_tot_ after heating was observed (Figure 7, Table 2).

## 4. Discussion

### 4.1. Chemical Pattern of Tinctures

Since THC-type varieties can be assumed as the common source for traditional tinctures, simple lab-scale maceration in this study simulates traditional preparation. Yet modern indoor chemotypes like ‘Northern lights’ here contain more THC_tot_ in comparison to the standard plants available in the UK around 40–150 years ago when still in official medicinal use [18,19,20]. Concentrations described here may therefore not have been reached with traditional, but rather with current illicit ‘homemade’ tinctures as occasionally recommended in the internet [21]. Further variation presumably existed regarding the purity of the starting material, i.e., the actual proportion of female flowers (plus adjacent leaves and smaller stalks) versus the leaves of lower plant parts, stalks, and seeds. The maximum case of ‘pure leaves’ (*Cannabis folium*) contained here approximately ten-fold less CAN_tot_ than pure *Cannabis flos*. Leaf extracts also showed qualitative discrepancies such as the flavonoid spectrum or the degree of cannabinoid decarboxylation.

The results confirm the previously reported observation that CBGA + CBG are the main co-cannabinoids besides THCA + THC in THC-type drugs, which has also been shown by other authors [13,22]. The own pharmacological properties of CBD and medicinal use of CBD varieties, but possibly more the still prevailing forensic purpose to distinguish between illicit psychoactive THC-type and non-psychoactive CBD-type (such as oil and fiber hemp varieties) might also be the reason that recent analytical approaches still concentrate on CBD, although marginally found in THC-type drugs, while CBG is not determined (e.g., [23]). It shows that the analytical objective of pharmaceutical quality control may require focus on compounds other than in forensics.

If the starting material consists strictly of female flowering tops, traditional tinctures using a minimum of 60% EtOH had a cannabinoid content > 6 mg/mL with THC_tot_ representing 70%–95% of the detected cannabinoids. The ratio to the main co-cannabinoids (CBG_tot_) to other cannabinoids, and to flavonoids was around 10:1, respectively. Therefore, any traditional use may be primarily linked to the presence of THCA, THC, and eventually their degradation compounds including CBN. This predominance of THC_tot_ is diminished by higher leaf portions in the drug, more polar extraction (e.g., 20% or 40% EtOH) as well as the use of ‘old’ drugs and tinctures. Cannabinoid and phenolic co-constituents may then influence overall effects: in a 40% leaf tincture the CAN_tot_/TPC ratio was only 0.2; in a 15-month-old 60% flower tincture, CBG_tot_ was half as concentrated as THC_tot_ representing more than 20% of all cannabinoids. Overall, cannabinoids and cannflavins were best extracted with 60%–90% EtOH from *flos*; other phenolics with 60% EtOH were from *folium*. Due to comparable polarity, cannflavins remained in their natural subordinated proportion versus cannabinoids (maximum around 1:50 in 60% EtOH tinctures from the flowers). Cannflavins were relatively stable in tinctures, although after 15 months additional flavonoid peaks appeared in line with observations from the reference substances as described under “Experimental”.

### 4.2. Stability of Dry Drugs and Liquid Extracts

The stability of cannabis, its derived products, and THC have been investigated in the 70s and 80s with focus on the forensic analysis. The oxidation of THC to CBN has been described earlier and storage under nitrogen protected from light was recommended [24,25,26]. Soon it was found that CBN was not the only decomposition product and it was found unstable upon light exposure, though less than THC or CBD [27,28]. Controversial reports on the stability of cannabinoids—in particular CBD—in chloroform resulted in recommendations for ethanolic solutions to be stored in the dark [29,30,31]. The prevailing post-harvest decomposition from the original acids to their neutral forms was by then not yet fully considered due to gas chromatographic detection of neutral cannabinoids only. This decarboxylation was found to be strongly accelerated by light, but occurring moderately at −18 °C in the dark [31] which has been recently confirmed at −25 °C for four months [32]. Zoller et al. [33] investigated the stability of THCA standard solutions and favoured neutral solutions at −20 °C to guarantee stability up to three months (in contrast THC > 1 year). Harvey [34] supposed that THCA seems to be more stable in dry plant samples as he still found it in 90-year-old cannabis samples in higher amounts than THC, although CBN and CBNA were the dominant compounds. Also, Turner and Fairbain [18,35] favoured the storage of dry drug samples rather than those in ethanolic solution. A recent study with dry samples (12 months at 25 °C protected from light) seems to support this with moderate changes only [32]. Although results obtained here showed no major qualitative difference between 40% tinctures from 5 and 24-month-old *Cannabis flos*, there was a substantial quantitative difference. It suggests that absolute THC_tot_ and CAN_tot_ are the parameters of concern during both liquid storage and dry storage (to a lesser extent), while CANA/CAN ratios are more affected in ethanolic solutions. Because neither 19 months dry (room temperature) nor 15 months liquid storage (EtOH, fridge) showed a fundamental increase in CBN—usually the standard marker for THC and cannabis degradation—any CBN values higher than 2% of the THC demonstrate more serious stability issues or older age. After 2 years of storage, a higher CBN proportion in the dry leaf than the dry flower drug was noted to an extent not developing over 15 months of liquid storage.

The stability test aimed to simulate realistic tincture storage conditions without intention to follow ICH pharmaceutical testing schemes or distinguishing between the influence of light and temperature. Complete dark conditions may not be assured in ‘real life’: even if sold in a carton covering box (presumably not used traditionally), it cannot be guaranteed that patients keep tincture bottles there. After 3 months, tests with ‘shelf’-stored tinctures were discontinued due to the progressed decarboxylation and overall reduction of THC_tot_. After 9 months in the ‘fridge’, THC increased, THCA lessened, and CBN slightly increased, but their sum was reduced pointing to other degradation processes. After 15 months, THC levels had dropped again, yet still above those of the fresh tinctures. These processes affected the absolute CAN_tot_ value of 60% and 90% tinctures more than that of the 40% tincture. The sum of CBGA and CBG remained almost constant. Interestingly, CBGA was clearly less decarboxylated in 40% than in 60% and 90% EtOH. For leaf-derived extracts, the decarboxylation processes as well as THC_tot_ and CAN_tot_ losses over 15 months in the ‘fridge’ were less pronounced compared to the flower extracts. The leaf drug had already advanced THCA degradation with higher THC and CBN levels at the starting point. CBN levels hardly increased. These results confirm previous investigations on the amounts of THCA, THC, and CBN and their sum [17]. It could be interesting whether for longer storage, a THCA:THC balance of 1:1 to 1:1.5 (despite absolute reduction) would be maintained as suggested by the data here.

### 4.3. Influence of Heating

The temperature impact on the decarboxylation was first shown by Turner & Mahlberg [16] where the drying at 37 °C compared to 60 °C yielded higher acid levels. The accelerated decarboxylation was found to start above 90 °C. As the optimum temperature for the decarboxylation of THCA to THC, Dussy et al. [17] proposed 150 °C (70% yield).

In this study, pasteurization of tinctures at 70 °C for two hours or heating at 80 °C for 20 min caused up to a 20% reduction in CAN_tot_ or THC_tot_ values, but changes were mostly below 10%. Heating had no effect on decarboxylation and changed only moderately overall tincture patterns. These qualitative and quantitative changes may be tolerable if treatments with increased temperature are necessary for production purposes. Together with the 3 months of comparison between shelf vs. fridge, this is in line with early reports where primarily light more than temperature (below 65 °C) and exposure to oxygen were found as critical factors [35,36].

## 5. Conclusions

This study provides a data set to estimate variations in traditional cannabis tincture quality and shows the determination via conventional HPLC/DAD. Alongside solvent polarity and tincture storage conditions, the age and quality of the original drug determines qualitative and quantitative tincture profiles. While the quality assurance in the herbal drug production has meanwhile reached industrial level [37], this systematic study shows the additional challenges at the drug preparation level for liquid extracts. Specifications of tinctures should include information on the original drug with distinction between *Cannabis folium* and *Cannabis flos,* with limitations of the leaf and stalk percentage in the latter and the flower percentage in the former.

In view of the considerable and variable content in cannabinoid acids and the low CBN content in relatively fresh tinctures, the absolute group markers THC_tot_ or CAN_tot_ and the ratio markers CANA/CAN (THCA/THC, CBGA/CBG) appear to be better stability markers than the conventional analytical targets THC, CBN, or THC/CBN ratios alone. However, determination of the acids may become superfluous e.g., after intended initial exhaustive decarboxylation via heat treatment over 110 °C. Contrary, pasteurization up to 80 °C does not lead to major deteriorations. In any case, storage without any stabilization is problematic: even when stored in the fridge, THC_tot_ values already reach after 3 months the 5% threshold that is usually considered acceptable in licensed herbal medicines (EMA/HMPC/41500/2010) [38]. Use and storage of non-standardized and non-stabilized traditional cannabis tinctures have therefore to be seen as critical because a consistent content in psychoactive and other cannabinoids cannot be guaranteed over time.

## Figures and Tables

**Table 1:** Group and ratio markers for cannabis extract characterisation. Description, relevance, and determination from high-performance liquid chromatography (HPLC) cannabinoid profile (CP) or fingerprint (FP) methods.

Marker	Calculation	Calculated as	Description/Relevance
THC_tot_	Σ THC, THCA, CBN	THC, THCA, CBN (*CP*/*FP*)	Total THC (‘potency marker’) - dominant in classic plants and preparations - constituent with highest CB1/CB2 receptor activity plus its unstable parent and main degradation product - psychotropic with legal/forensic importance
CBD_tot_	Σ CBD, CBDA	CBD (*CP*)	Sum of CBD and CBDA - dominant in some chemotypes (intermediate, hemp) - main constituents without CB1/CB2 receptor affinity - non-psychotropic but specific pharmacological effects
CBG_tot_	Σ CBG, CBGA	CBG (*CP*)	Sum of CBG and CBGA - dominant in some chemotypes - main constituents without CB1/CB2 receptor affinity - non-psychotropic but specific pharmacological effects
CAN	Σ neutral cannabinoids	THC, CBD, CBG, CBN (*CP*/*FP*)	Neutral (decarboxylated) cannabinoids - dominant upon excessive drying, heating, age
CANA	Σ acidic cannabinoids	THC, CBD, CBG, CBN (*CP*/*FP*)	Acidic (carboxylated) cannabinoids - dominant in fresh plants and preparations without heating
CAN_tot_	Σ neutral and acidic cannabinoids, CAN + CANA	THC, CBD, CBG, CBN (*CP*/*FP*)	Total cannabinoid content
oCAN	CAN_tot_ − (Σ THC_tot_ + CBD_tot_ + CBG_tot_)	THC (*CP*)	Other cannabinoids than those usually found as main cannabinoids in common chemotypes, (i.e., THC_tot_, CBD_tot_, CBG_tot_)
CFL	CFL-A + CFL-B	CFL-A, CFL-B (*CP*)	Cannflavins (cannabis specific prenylated flavones)
TPC	Σ flavonoids and phenolcarbonic acids	vitexin, chlorogenic acid (*FP*)	Total phenolic content (without CFL)
THC_tot_/(CBD_tot_ + CBG_tot_)	Σ THC, THCA, CBN/Σ CBD_tot_, CBG_tot_	as for THC_tot_, CBG_tot_, CBD_tot_ (*CP*/*FP*)	‘chemotype marker’ - ratio main constituents in common chemotypes - ratio main CB1/CB2 active vs. inactive constituents (plus acidic pro-drug) - ratio main psychotropic/non-psychotropic constituents (plus acidic pro-drug) - legal/forensic importance
CANA/CAN	Σ CANA/Σ CAN	as for CANA, CAN (*CP/FP*)	‘decarboxylation marker’ - ratio acidic /neutral cannabinoids - indicator for drug and extract quality and age
CAN_tot_/TPC	CAN_tot_/TPC	as for CAN_tot_, TPC (*FP*)	‘polarity marker’ - indicator for extract/solvent polarity - indicator for leave and flower portions - potential influence of phenolics on cannabinoid activity

Neutral cannabinoids (CAN); cannabinoid acids (CANA); total cannabinoids (CAN_tot_); cannabidiol (CBD); cannabidiolic acid (CBDA); cannabidiol + cannabidiolic acid (CBD_tot_); cannabigerol (CBG); cannabigerolic acid (CBGA); cannabigerol + cannabigerolic acid (CBG_tot_); cannabinol (CBN); cannflavin A (CFL-A); cannflavin B (CFL-B); other cannabinoids (oCAN); Δ9-tetrahydrocannabinol (THC); Δ9-tetrahydrocannabinolic acid A (THCA); Δ9-tetrahydrocannabinol + Δ9-tetrahydrocannabinolic acid A + cannabinol (THC_tot_); total phenolic content (TPC).

**Table 2:** Relative changes (value in fresh tincture set as 100% except CANA/CAN ratios) of stability-indicating key markers in ethanolic tinctures from Cannabis flos (F40, F90) according to storage and additional heating.

Marker		Fresh	3 Months ‘Shelf’	3 Months ‘Fridge’	3 Months ‘Fridge’	3 Months ‘Fridge’	9 Months ‘Fridge’	15 Months ‘Fridge’
					+2 h 70 °C	+20 min 80 °C		
F40	THC (%)	100.0	193.5	119.5	73.5	72.2	229.9	179.2
	CBN (%)	(<LoQ) ^1^	(0.01 mg/mL)	(0.005 mg/mL)	(<LoQ) ^1^	(<LoQ) ^1^	(0.009 mg/mL)	(0.005 mg/mL)
	THC_tot_ (%)	100.0	85.1	78.3	66.0	62.6	70.5	60.1
	CAN_tot_ (%)	100.0	90.6	84.8	78.4	69.2	85.1	77.7
	CANA/CAN	5.72	1.57	3.31	5.75	5.51	1.22	1.52
F90	THC (%)	100.0	175.0	121.4	118.1	126.7	194.4	102.6
	CBN (%)	100.0	118.7	102.7	101.9	105.5	118.7	146.4
	THC_tot_ (%)	100.0	84.5	100.5	93.3	91.4	98.2	55.2
	CAN_tot_ (%)	100.0	90.1	100.5	97.6	96.8	100.8	70.8
	CANA/CAN	1.97	0.48	1.49	1.39	1.20	0.57	0.53

^1^ Limit of quantification 0.005 mg/mL.

## Abbreviations

The following abbreviations are used in this manuscript:

CAN	neutral cannabinoids
CANA	cannabinoid acids
CAN_tot_	total cannabinoids (CAN + CANA)
CBC	cannabichromene (CAS 20675-51-8)
CBD	cannabidiol (CAS 13956-29-1)
CBDA	cannabidiolic acid (CAS 1244-58-2)
CBD_tot_	cannabidiol + cannabidiolic acid
CBG	cannabigerol (CAS 25654-31-3)
CBGA	cannabigerolic acid (CAS 25555-57-1)
CBG_tot_	cannabigerol + cannabigerolic acid
CBN	cannabinol (CAS 521-35-7)
CFL-A	cannflavin A (CAS 97502-36-8)
CFL-B	cannflavin B (CAS 76735-58-5)
oCAN	other cannabinoids (CAN_tot_—THC_tot_—CBD_tot_—CBG_tot_)
THC	Δ9-tetrahydrocannabinol (CAS 1972-08-3)
THCA	Δ9-tetrahydrocannabinolic acid A (CAS 23978-85-0)
THC_tot_	Δ9-tetrahydrocannabinol + Δ9-tetrahydrocannabinolic acid A + cannabinol
TPC	total phenolic content
